# Coexistent papillary thyroid carcinoma diagnosed in surgically treated patients for primary versus secondary hyperparathyroidism: same incidence, different characteristics

**DOI:** 10.1186/s12893-019-0556-y

**Published:** 2019-07-16

**Authors:** Cristina Preda, Dumitru Branisteanu, Ioana Armasu, Radu Danila, Cristian Velicescu, Delia Ciobanu, Adrian Covic, Alexandru Grigorovici

**Affiliations:** 1 Faculty of Medicine, Department of Endocrinology, “Gr igore T Popa” University of Medicine and Pharmacy, 16 Universitatii Str, 700115 Iasi, Romania; 2 Faculty of Medicine, Department of Surgery, “Grigore T Popa” University of Medicine and Pharmacy, 16 Universitatii Str, 700115 Iasi, Romania; 3 Faculty of Medicine, Department of Morphopathology, “Grigore T Popa” University of Medicine and Pharmacy, 16 Universitatii Str, 700115 Iasi, Romania; 4 Faculty of Medicine, Department of Nephrology, “Grigore T Popa” University of Medicine and Pharmacy, 16 Universitatii Str, 700115 Iasi, Romania; 5 Academy of Romanian Scientists, Bucuresti, Romania; 6 Department of Morphofunctional Sciences, “Grigore T. Popa” University of Medicine, Iasi, Romania

**Keywords:** Primary hyperparathyroidism, Secondary hyperparathyroidism, Papillary thyroid carcinoma, Association

## Abstract

**Background:**

The coexistence of hyperparathyroidism and thyroid cancer presents important diagnostic and management challenges. With minimally invasive parathyroid surgery trending, preoperative thyroid imaging becomes more important as concomitant thyroid and parathyroid lesions are reported. The aim of the study was to evaluate the rate of thyroid cancer in patients operated for either primary (PHPT) or secondary hyperparathyroidism (SHPT).

**Methods:**

Our retrospective study included PHPT and SHPT patients submitted to parathyroidectomy and, when indicated, concomitant thyroid surgery between 2010 and 2017.

**Results:**

Parathyroidectomy was performed in 217 patients: 140 (64.5%) for PHPT and 77 (35.5%) for SHPT. Concomitant thyroid surgery was performed in 75 patients with PHPT (53.6%), and 19 papillary thyroid carcinomas (PTC) were found, accounting for 13.6% from all cases with PHPT and 25.3% from PHPT cases with concomitant thyroid surgery. Thirty-one of operated SHPT patients (40.3%) also underwent thyroid surgery and 9 PTC cases were diagnosed (11.7% of all SHPT patients and 29% of patients with concomitant thyroid surgery).

We found differences between PHPT and SHPT patients (*p* < 0.001) with respect to age (54.6 ± 13y versus 48.8 ± 12y), female-to-male ratio (8:1 versus ~ 1:1), surgical technique (single gland parathyroidectomy in 82.8% PHPT cases; versus subtotal parathyroidectomy in 85.7% SHPT cases) and presurgical PTH (357.51 ± 38.11 pg/ml versus 1020 ± 161.38 pg/ml).

Morphopathological particularities, TNM classification and multifocality incidence of PTC were similar in the two groups. All PTC from patients with SHPT were thyroid microcarcinomas (TMC, i.e. tumors with a diameter smaller than 1 cm), whereas seven out of the 19 cases with PTC and PHPT were larger than 1 cm.

**Conclusions:**

PTC was frequently and similarly associated with both PHPT and SHPT irrespective of presurgical PTH levels. Thyroid tumors above 1 cm were found only in patients with PHPT. Investigators should focus also on associated thyroid nodular pathology in patients with PHPT.

## Background

Primary hyperparathyroidism (PHPT) has a prevalence of 0.1 to 0.4% in the general population, being more frequently diagnosed in the fifth decade of life, in female patients [1–4]. Several reports described an increased incidence risk of cancer in patients with PHPT [5–8].

The association of thyroid disease and PHPT was first described more than 70 years ago [9]. More recent reports evaluated the necessity and the extent of thyroid investigation prior to parathyroid surgery [10]. Patients with secondary (SHPT) and tertiary hyperparathyroidism (parathyroid gland autonomization) were also described to associate thyroid nodular disease and cancer [10–14]. Present literature does not include, however, studies comparing PHPT and SHPT with respect to their association with thyroid cancer.

## Methods

*The aim* of our study was to characterize and compare the frequency of thyroid cancer among patients with primary or secondary hyperparathyroidism, who underwent both parathyroidectomy and thyroid surgery in a single surgical center.

### Design and setting

**Fig. 1:**
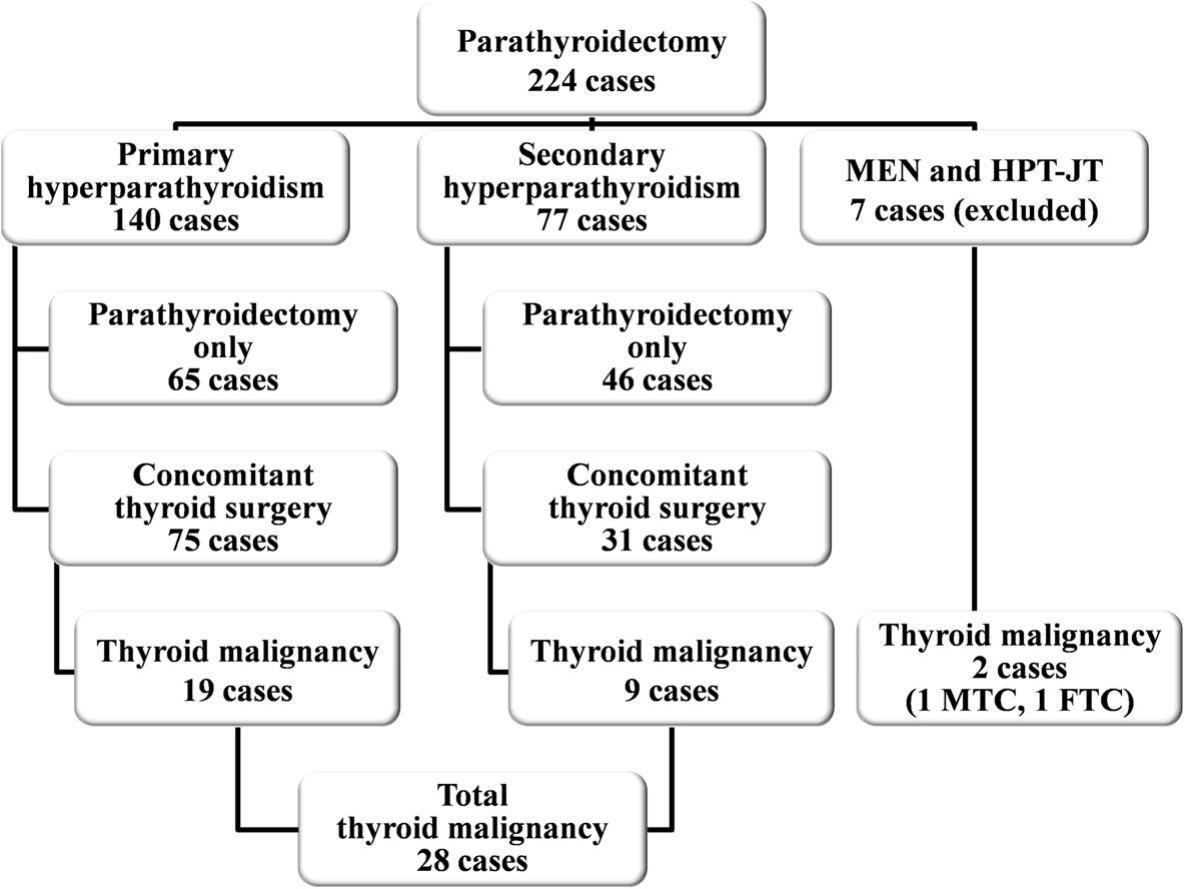
Group stratification according to the type of hyperparathyroidism, single HPT vs. concomitant thyroid disease and thyroid malignancy found after surgery

After receiving approval from the institutional Ethics Committee, we reviewed retrospectively all parathyroidectomies performed between January 2010 and December 2017 at the Department of General Surgery, “Sf. Spiridon” Emergency Hospital Iasi. We included an initial number of 224 patients diagnosed and surgically treated for PHPT or SHPT. PHPT cases were diagnosed in the Endocrinology Department of “Sf. Spiridon” Emergency Hospital Iasi whereas SHPT cases in the context of advanced chronic kidney disease (CKD) were addressed from the Department of Nephrology, “C.I. Parhon” University Hospital Iasi. Surgery was performed by a team specialized in endocrine pathology. Patients’ clinical data and morphopathological results were documented from the hospital’s electronic registry. Seven patients had hyperparathyroidism in syndromic conditions: 6 cases of multiple endocrine neoplasia syndromes (MEN), 1 case of hypeparathyroidism - jaw tumor syndrome (HPT-JT), and were excluded from the study. The remaining 217 patients assigned to the main study group included 140 patients diagnosed with PHPT (64.5%) and 77 patients diagnosed with SHPT in the condition of advanced chronic kidney disease (35.5%). Parathyroidectomy was accompanied by thyroidectomy in 75 out of the 140 patients with PHPT and 31 out of the 77 patients with SHPT (Fig. 1). The main reason for thyroidectomy was the coexistence of solitary or multiple thyroid nodules, discovered before surgery and accompanied by compressive or esthetical complaints, followed by the incidental intraoperative findings. FNAB was performed from large thyroid nodules in 28 patients with PHPT and 9 patients with SHPT, but no elements of suspicion for malignancy were found before surgery. Surgical intervention on thyroid varied function of the dimensions and spreading of the observed lesions from adenomectomy and/or hemithyroidectomy to subtotal or total thyroidectomy.

*The characteristics* of patients from the PHPT and SHPT groups are depicted in Table 1 and Table 2. All 28 patients diagnosed with PTC underwent thyroid surgery for the coexistence of thyroid macronodularization and the diagnosis of PTC came as an incidental histological finding.

**Table 1:** Comparison of parathyroid and concomitant thyroid disease in patients with PHPT vs. SHPT

		*PHPT*		*SHPT*		*p*-*Value*
		*No. cases*	*(%)*	*No. cases*	*(%)*	
General data	Number of patients	**140**	(64.5)	**77**	(35.5)	
	Mean age	54.6 ± 13		48.8 ± 12		0.001
	Sex					< 0.001
	Male	15		39		
	Female	125		38		
	Female:male ratio	8:1		~ 1:1		
	Preoperative PTH level [pg/ml]	357.51 ± 38.11		1020 ± 161.38		< 0.001
Parathyroid disease	Parathyroid surgery*					< 0.001
	Single gland parathyroidectomy	116	(82.8)	7	(9.1)	
	*Right superior gland*	*17*	*(14.7)*	*0*	*(0)*	
	*Right inferior gland*	*11*	*(9.5)*	*1*	*(14.3)*	
	*Left superior gland*	*47*	*(40.5)*	*1*	*(14.3)*	
	*Left inferior gland*	*38*	*(32.8)*	*2*	*(28.6)*	
	*Ectopic*	*3*	*(2.5)*	*0*	*(0)*	
	*Unspecified*	*0*	*(0)*	*3*	*(42.8)*	
	Two glands parathyroidectomy	8	(5.7)	2	(2.6)	
	Subtotal parathyroidectomy	10	(7.2)	66	(85.7)	
	Multiple parathyroid surgeries	6	(4.3)	2	(2.6)	
	Parathyroid morphopatology results*					< 0.001
	Parathyroid carcinoma	1	(0.7)	–	–	
	Parathyroid adenoma	92	(65.7)	3	(3.9)	
	Adenomatous hyperplasia	47	(33.6)	74	(96.1)	
	*Nodular*	*27*	*(57.4)*	*59*	*(79.7)*	
	*Diffuse*	*7*	*(14.9)*	*3*	*(4.1)*	
	*Both nodular and diffuse*	*8*	*(17.1)*	*9*	*(12.1)*	
	*Unspecified*	*5*	*(10.6)*	*3*	*(4.1)*	
Thyroid disease	Thyroid surgery	**75**	(53.6*)	**31**	(40.3*)	0.061
	Adenomectomy	4	(5.3**)	5	(16.1**)	
	Lobectomy/Lobisthmectomy	21	(28**)	11	(35.5**)	
	*Right*	*7*	(33.3**)	*6*	(63.6**)	
	*Left*	*14*	(66.7**)	*4*	(36.4**)	
	Subtotal thyroidectomy	1	(0.7**)	–	–	
	Total thyroidectomy	48	(34.3**)	14	(45.6**)	
	Multiple thyroid surgeries	1	(0.7**)	2	(2.6**)	
	Thyroid morphopathology result					
	*Papillary thyroid carcinoma (PTC)*	**19**	(13.6*) (25.3**)	**9**	(11.7*) (29**)	0.692 0.694
	*Benign lesions* Thyroid adenoma	9	(12**)	–	–	
	Adenomatous/colloid goiter	14	(18.7**)	14	(45.2**)	
	Adenomatous/colloid goiter with adenomas	40	(53.3**)	10	(32.3**)	
	Hashimoto thyroiditis with nodularization	12	(16**)	6	(19.4**)	
	Graves Basedow disease	–	–	1	(3.2**)	

*percentage of PHPT or SHPT cases, respectively; **percentage of concomitant disease cases

**Table 2:** Particularities of papillary thyroid carcinoma associated with PHPT or SHPT

	PHPT		SHPT		*p*
	No. cases	(%)	No. cases	(%)	
Papillary thyroid carcinoma (PTC)	**19**		**9**		0.517
Classic variant of PTC	14	(73.6)	8	(88. 9)	
Follicular variant of PTC	4	(21.1)	1	(11.1)	
Oncocytic variant of PTC	1	(5.3)	–	–	
T (tumor) classification					
T1 (a and b)	13	(68.4)	9	(100)	
T2	–	–	–	–	
T3 (a and b)	6	(31.6)	–	–	
T4	–	–	–	–	
N (lymph node) classification					0.466
Nx	13	(68.4)	4	(55.6)	
N0	5	(26.3)	5	(44.4)	
N1	1	(5.3)	–	–	
Maximum tumor diameter					
< 10 mm (microcarcinoma)	12	(63.1)	9	(100)	
≥ 10 and < 20 mm	3	(15.8)	–	–	
≥ 20 and < 40 mm	1	(5.3)	–	–	
≥ 40 cm	3	(15.8)	–	–	
Mean tumor size [mm]	13.6 ± 18.4		2.7 ± 2.1		0.005
Multifocality	4	(21.1)	1	(11.1)	0.507

*Statistical analysis* was performed using SPSS 20.0 software (IBM Corp., Armonk, NY, USA). Descriptive statistics for continuous variables was expressed as mean ± standard deviation. Bivariate analysis was conducted with independent sample t-test, comparing means. Categorical variables were expressed in number and percent (%), and Fisher’s chi-square test was used to assess the differences between groups with regard to the categorical variables.

## Results

### Primary hyperparathyroidism

Mean age of patients with PHPT that underwent parathyroidectomy was 54.6 ± 13 years (between 18 and 77). The studied group consisted of 125 female and 15 male patients determining a female-to-male ratio of 8:1. PHPT was more frequently diagnosed in the fifth and sixth decade of life, similarly in men and women. The most frequent initial type of surgery performed in patients with PHPT was the minimally invasive parathyroidectomy (116 cases, 82.8%). Minimal intervention was however converted to open neck surgery due to the coexistence of important thyroid lesions found during operation in 26 cases, whereas in 25 cases of presurgically diagnosed nodular thyroid pathology, limited ipsilateral thyroid intervention was performed without conversion. Other 24 patients with PHPT and coexistent presurgically diagnosed large thyroid nodularization were thyroidectomized by open neck surgery together with the excision of diseased parathyroid tissue. Hyperparathyroidism affected more often the left parathyroid glands: 47 (33.6%) of surgical interventions concerned the left superior parathyroid gland and 38 (27.1%) the left inferior parathyroid gland. Morphopathological examination confirmed the diagnosis of parathyroid adenoma in 92 cases (65.7%) and of hyperplasia in 47 cases (33.6%). One patient (0.7%) in this group was diagnosed with parathyroid carcinoma. Thyroid surgery was performed in 75 cases (53.6%) because of the presence of associated thyroid pathology, mainly thyroid nodules. The most frequent procedure was total thyroidectomy performed in 48 cases (34.3%), followed by lobectomy/lobisthmectomy in 21 cases (28%). The most frequent pathology described was adenomatous/colloid goiter with and without nodularization in 38 patients (27.1%); followed by Hashimoto thyroiditis with nodularization in 10 cases (7.1%) and solitary adenomas in 8 cases (5.7%, Table 1). Morphopathological investigation diagnosed 19 cases of PTC, which accounted for 13.6% of all PHPT cases and 25.3% of patients who underwent both thyroid and parathyroid surgery. Micropapillary tumors (TMC) were detected in 12 cases (63.2%) whereas in the other 7 cases differentiated thyroid carcinomas had a diameter above 1 cm. Lymph node invasion was documented in one case (5.3%) and multifocal tumors were present in 4 patients (21.1%). The predominant histological variant was the classical PTC in 14 cases (73.6%), followed by the follicular variant of PTC in 4 cases (21.1%) and the oncocytic variant in 1 case (5.3%, Table 2).

### Secondary hyperparathyroidism

The mean age of patients with SHPT that underwent parathyroidectomy was 48.8 ± 12 years (between 25 and 77 years). Sex distribution was balanced, with a female-to-male ratio of ~ 1:1 (39 male patients, 38 female patients, Table 1). Operated men were younger (between 30 and 50 years old), while women operated for SHPT were in the fifth decade. The elected procedure in SHPT was subtotal parathyroidectomy performed in 66 cases (84.4%), as parathyroid hyperplasia was the most frequent diagnosed pathology (74 cases, 96.1%). Thirty-one (40.3%) patients underwent both thyroid and parathyroid surgery leading to the diagnosis of 9 cases with PTC (8 cases of classic variant and 1 case of follicular variant of PTC), which accounted for 11.7% of all patients diagnosed with SHPT and of 25.7% of patients who underwent both thyroid and parathyroid surgery. All tumors were TMC, and in 8 out of 9 cases the lesions were unifocal. Similarly with PHPT patients the most frequent thyroid pathology was the adenomatous/colloid goiter with and without nodularization in 16 cases (51.7%, Table 2).

### Comparison between patients with PHPT and SHPT

We found significant age (*p* = 0.001) and sex distribution (*p* < 0.001) differences, as well as type of parathyroid surgery, with more frequent minimally invasive surgery in PHPT (where the disease frequently involves only one parathyroid gland) and more frequent open neck surgery in SHPT, usually accompanied by hyperplasia of all parathyroid glands (*p* < 0.001, Table 1). There were no significant differences between the type of thyroid surgery (*p* = 0.109) performed, as the most frequent procedure was total thyroidectomy in both PHPT and SHPT. Mean presurgical PTH levels were higher in patients with SHPT than with PHPT (357.5 ± 38.1 pg/ml in PHPT and 1020 ± 161.4 pg/ml in SHPT, p < 0.001, Table 1). Morphopathological examination revealed more frequent solitary parathyroid adenoma in PHPT and more frequent multiglandular hyperplasia in SHPT (p < 0.001). The incidence of PTC was not different in patients with PHPT and SHPT (*p* = 0.694, Table 1). We did not find significant differences between the two groups with respect to the histological particularities of papillary carcinoma (*p* = 0.517), TNM classification (lymph node status: *p* = 0.253; no distant metastases involved), tumor diameter (*p* = 0.571) and multifocality (*p* = 0.507, Table 2). PTC with a diameter larger than 1 cm were found only in 6 patients with PHPT but at none of the patients with SHPT.

## Discussion

PHPT is considered nowadays a frequent disease, mainly due to the high incidence of mild sporadic forms appearing more often in women between 50 and 60 years of age [1, 3, 15]. CKD is also a frequent disease appearing equally in males and females. Advancing CKD is often accompanied by reactive SHPT which appears at an earlier age than PHPT [16]. Due to deleterious effects of very high PTH levels on bone (renal osteodystrophy), selected patients with SHPT need parathyroid gland removal as an ultimate attempt to arrest this severe complication [16].

Most literature studies focused on the association between parathyroid and thyroid surgery only in cases of PHPT [17–31] or SHPT [12, 15, 32] alone, and just a few dealt with this association in both PHPT and SHPT operated cases taken together [33, 34]. These studies reported a wide rate range of concomitant parathyroid and thyroid intervention (between 2.5 and 84.3%) [12, 15, 17–33].

Our study specifically evaluated the association of thyroid pathology in parathyroidectomized patients. We included 140 patients diagnosed with PHPT and 77 cases with advanced CKD and SHPT requiring parathyroidectomy. Seventy-five patients with PHPT and 31 patients with SHPT (53.6 and 40.3% respectively) underwent also thyroid surgery, mainly decided for nodular thyroid enlargement.

Patients with PHPT had a more advanced diagnostic age, predominance of females and of solitary parathyroid adenomas. Solitary parathyroid disease implied a more frequent use of minimally invasive parathyroidectomy at PHPT patients, whereas open neck surgery was the elective technique used in patients with SHPT, where patients are more at risk for parathyroid hyperplasia. Contrary to other reports, in our patients with PHPT we found parathyroid adenomas mainly on the left side (in 73.3% cases). Whereas one previous study was concluded more than four decades ago, when the only surgical technique used was open neck surgery [35], the other study was performed recently but on a geographically distinct population [36], therefore genetic variations may be the substrate of different adenoma locations.

The most frequent type of thyroid surgery performed concomitantly in HPT was reported to be either total/subtotal thyroidectomy [17, 37] or unilateral lobisthmectomy [33]. Total thyroidectomy was commonly used in our study, justified by the high frequency of nodular disease in a region previously known for iodine deficient disorders [38]. The most frequent histological findings described in the literature are nodular goiter (8.4–47% of HPT cases), followed by thyroiditis (1.4–17.6%) and solitary thyroid adenoma (3.8–6.4%) [18–22]. Graves’ disease remains an infrequent association (1.8%) [19]. Our findings were in accordance with these previous results.

Parathyroid carcinoma was diagnosed in only one case of PHPT, and it did not associate any thyroid disease. Other studies also described an incidence of parathyroid carcinoma in less than 1% of PHPT cases [39]. The association of parathyroid cancer and differentiated thyroid cancer was rarely described, suggesting coincidence rather than causality [40–43].

Non-medullary thyroid cancer was discovered with an incidence varying from 0.9 to 18.2% (mean value of 4%) of surgically treated PHPT patients. Autopsy controlled studies showed that thyroid cancer occurs more frequently in patients with PHPT, fact not observed for autoimmune or thyroid nodular disease [44, 45]. PHPT patients seem to have an increased overall cancer risk and parathyroidectomy is not a risk-reducing, but rather a delaying factor in the occurrence of cancer [7]. We found PTC in 19 (13.6%) of our PHPT cases - third highest rate of occurrence reported (Table 3). In accordance with previous reports [17, 33, 46–48], twelve out of 19 cases (63.2%) were TMC and did not involve any lymph node or distant metastases. Low risk TMC could be managed with lobectomy instead of total thyroidectomy [49–51], improving the quality of life. Rare metastatic TMC cases were however anecdotally described [52].

**Table 3:** Frequency of thyroid cancer in patients undergoing parathyroidectomy

*Study*	*Country*	*Year*	*(P)HPT patients*	*Concomitant thyroid cancer*	
			*No.*	*No.*	*%*
Masatsuguet al (21)	Japan	2005	110	20	18.2
Kösem M et al (22)	Turkey	2004	51	9	17.6
Current study	Romania	2019	140	19	13.6
Attie J and Vardhan R (26)	USA	1993	242	31	12.8
Gates JD et al (27)	USA	2009	24	3	12.5
Kutlutürk K et al(19)	Turkey	2014	46	5	10.9
Trout HH and Moulder DJ(28)	USA	1972	30	3	10.0
Ellenberget al(29)	USA	1962	93	7	7.5
Wright MC et al(82)	USA	2017	103	7	6.8
Livolsi VA and Feind CR(54)	USA	1976	471	31	6.6
Kaplan et al(45)	USA	1971	62	4	6.5
Krementz et al(34)	USA	1971	100	6	6.0
Morita SY et al (49)	USA	2008	200	12	6.0
Petro AB and Hardy JD(88)	USA	1974	104	5	4.8
Monroe DP et al (30)	USA	2008	194	9	4.6
Sindhu S and Campbell P (24)	Australia	2001	65	3	4.6
Prinzet al (55)	USA	1982	351	15	4.3
Krause U et al (31)	Germany	1991	163	6	3.7
Feind CR(32)	USA	1964	119	4	3.4
Nishiyamaet al (56)	Japan	1979	420	13	3.1
Beus KS and Stack BC (33)	USA	2003	101	3	3.0
Burmeisteret al (18)	USA	1996	700	18	2.6
Yazici P *et al* (89)	Turkey	2015	228	6	2.6
Jovanovic MD *et al* (17)*	Serbia	2017	849	21	2.5
Linos DA *et al* (16)	USA	1982	2058	51	2.5
Bentrem DJ *et al* (23)	USA	2002	580	12	2.1
ZhengYX*et al* (20)	China	2007	52	1	1.9
Ogburn P and Black B(10)	USA	1956	230	4	1.7
EmirikçiS *et al*(41)	Turkey	2015	550	5	0.9
*Total*			*8862*	*358*	*4*

Parathyroidectomy decreases mortality in patients with CKD and severe SHPT [53]. (Sub)total parathyroid excision is frequently needed, since all parathyroid glands are involved. Several authors described a more frequent association of CKD [32, 54, 55], chronic dialysis [56, 57], SHPT [12, 15, 58] or kidney transplant [14, 75, 77. 78] with thyroid cancer than in the general population. Although all these studies suggested that CKD is accompanied by an increased risk of malignancy, including PTC, they did not, however, systematically evaluate the patients with SHPT submitted to both parathyroidectomy and thyroidectomy. These patients are different than other patients with CKD, in terms of higher severity of parathyroid and bone disease.

We confirmed 9 cases of PTC out of the 31 SHPT patients also operated for goiter, all of them having infracentrimetric dimensions (TMC) while only one had multifocal lesions. Similarly, other studies confirmed a predominance of TMC, being attributed to a closer thyroid surveillance in the context of hyperparathyroidism [34]. A previous study in our center including 43 patients with SHPT who underwent parathyroidectomy between 1994 and 2004 and, when indicated, concomitant thyroid surgery (in 17 cases, 39.5% of SHPT), revealed a lower incidence of papillary TMC of only 4.7% (2 out of 17 cases) [59]. The participants were however fewer and TMC incidence was not significantly different from the present study (9 out of 31 cases, *p* = 0.125 by the Chi-square test).

PHPT and SHPT are different etiopathogenic entities, and literature data regarding the association between SHPT and thyroid cancer is scarce [10, 13, 33, 34]. Our study demonstrated for the first time that the incidence of PTC was high and similar in both PHPT and SHPT (13.6 and 11.7% respectively, *p* = 0.517) in patients operated in the same surgical center. Likewise, Burmeister et al. reported similar, albeit lower frequencies for both PHPT (2.6%) and SHPT (3.2%, *p* = 0.550) [34].

The opinions on the association of HPT with non-medullary thyroid cancer or thyroid nodularization shifted over time from mere coincidental [21, 23, 60, 61] to considering them causally related [17, 52, 62–64]. Although PHPT and concomitant medullary thyroid cancer (MTC) are well described in MEN2A syndrome, no obvious genetic link between PTC and PHPT has been yet demonstrated. Most cases of PHPT are sporadic and may associate a germline mutation in MEN1, CDC73, CASR, CDKIs or PTH genes, especially in patients under 45 [65]. PTC is the most common thyroid malignancy and mutations in RET proto-oncogene, BRAF and Ras may be involved in its development [66], especially in younger patients and after radiation exposure [67].

Some studies investigated several predisposing factors for PTC in PHPT, such as the tumor promoting effect of PTH [60], the goitrogenic effect and increased mitotic activity induced by hypercalcemia [7, 68, 69] and neck irradiation [7, 69, 70]. A presumed role for PTH excess in triggering the onset of PTC remains controversial [34]. One of the criteria for choosing parathyroidectomy in SHPT due to CKD is that of high PTH levels (over 800 pg/ml) [16], whereas even milder forms of PHPT with modestly elevated PTH actually represent an indication for surgery [15]. Not surprisingly, our patients with SHPT had therefore much higher pre-operatory PTH levels when compared to PHPT patients. Despite this clear quantitative difference, the incidence of PTC was similar in the two groups (25.3% in PHPT and 29% in SHPT, *p* = 0.694), suggesting that higher PTH may not increase PTC incidence further, and that PTH may therefore even not be involved at all in the onset of PTC. Moreover, the incidence of PTC drastically increased recently. This increase is exclusively due to the over-diagnosis of TMC frequently found with more detailed histological investigation, more often thyroid ultrasound investigation and ultrasound-guided fine needle aspiration biopsy (FNAB) [32, 54, 71–75]. The evolution of TMC is usually indolent and their radical therapy did not contribute to a decrease of mortality, therefore certain authors even recommend conservative therapy and follow-up in these cases [32, 55, 75]. The presence of TMC was also described in up to one third of thyroid gland autopsies of persons deceased for other reasons [58, 71, 73, 74]. Retrospective studies performed in Romania showed a higher incidence of thyroid cancer after the Chernobyl accident [65, 66]. The increase of thyroid carcinoma incidence in Romania is paralleling, however, the same observation worldwide, irrespective of radiation exposure and may be rather related to the higher sensitivity in the diagnosis of TMC [32, 54, 58, 71–75].

Since TMC incidence is so high in the general population, it is not unexpected to observe the presence of TMC also in patients operated for both thyroid and parathyroid glands, irrespective of parathyroid pathology. Six patients with PHPT, but no patients with SHPT had, however, PTC with a diameter larger than 1 cm, and of over 4 cm in 3 cases. These results suggest that larger forms of PTC with presumed poorer prognosis may be occasionally found into the thyroids of patients with PHPT, but less frequently in patients with SHPT. The coexistence of a nodular lesion in the thyroid of patients with PHPT, which is usually detectable before surgery during ultrasound investigation of the cervical anterior region should therefore not be neglected, FNAB being strongly advisable in order to rule out malignancy.

It is not clear how PHPT may influence the evolution of differentiated thyroid malignancy. As stated above, PTH levels seem not to be important since larger PTC were found exclusively in patients with PHPT, despite their much lower PTH compared to SHPT patients. A presumed common genetic background, although not yet defined, may thus predispose certain patients to both PHPT and PTC with larger dimensions.

Our study is the first to compare the incidence and histology of thyroid carcinomas between patients concomitantly operated in the same medical center for PHPT or SHPT caused by CKD, and coexistent thyroid pathology. The limitations of this study consist, however, in its retrospective nature, as well as the absence of a control group submitted to thyroid surgery, but without coexistent parathyroid pathology.

## Conclusions

PTCs are frequently diagnosed in association with HPT, especially in regions with endemic goiter. Undiagnosed concomitant thyroid nodules represent the main hazard to minimally invasive procedures for parathyroid adenomas, since they may veil malignancy [56, 57]. The incidence of PTC is similar in patients with PHPT and SHPT, even if PTH levels are significantly higher in patients operated for SHPT, suggesting that PTH may not be directly involved in the PTC pathogenesis. Although PTC found in patients with SHPT were all TMC, seven out of the 19 patients with PHPT and PTC had thyroid tumors with a diameter above 1 cm, and even above 4 cm in three cases. It is not clear why larger PTC are present in an important number of patients with PHPT, but not SHPT.

A particular genetic background may be involved in these patients, fact that should be further investigated. Associated thyroid nodular pathology in patients with PHPT should therefore not be neglected and FNAB of larger thyroid nodules should be routinely performed in these cases.

## Data Availability

The raw data supporting our findings which were used and/or analyzed during the current study are available in the Archive of the St Spiridon Hospital. These data can be requested from the corresponding author on reasonable request. Datasets were attached as supplemental material to the article.

## Abbreviations

CKD: Chronic kidney disease
FNAB: Fine needle aspiration biopsy
HPT: Hyperparathyroidism
PHPT: Primary hyperparathyroidism
PTC: Papillary thyroid carcinoma
PTH: Parathyroid hormone
SHPT: Secondary hyperparathyroidism
TMC: Thyroid microcarcinoma

## References

[1] Arrangoiz R, Cordera F, Lambreton F, De Leon EL, Moreno E (2016). Current thinking on primary hyperparathyroidism. JSM Head Neck Cancer Cases Rev.

[2] Gopinath P, Mihai R (2011). Hyperparathyroidism. Surgery..

[3] Wermers RA, Khosla S, Atkinson EJ, Achenbach SJ, Oberg AL, Grant CS (2006). Incidence of primary hyperparathyroidism in Rochester, Minnesota, 1993–2001: an update on the changing epidemiology of the disease. J Bone Miner Res.

[4] Silverberg SJ, Bilezikian JP (2004). Asymptomatic primary hyperparathyroidism: a medical perspective. Surg Clin North Am.

[5] Palmieri S, Roggero L, Cairoli E, Morelli V, Scillitani A, Chiodini I (2017). Occurrence of malignant neoplasia in patients with primary hyperparathyroidism. Eur J Intern Med.

[6] Goswami S, Ghosh S (2012). Hyperparathyroidism: cancer and mortality. Indian J Endocrinol Metab.

[7] Nilsson I-L, Zedenius J, Yin L, Ekbom A (2007). The association between primary hyperparathyroidism and malignancy: nationwide cohort analysis on cancer incidence after parathyroidectomy. Endocr Relat Cancer.

[8] Pickard Amy L., Gridley Gloria, Mellemkjae Lene, Johansen Christoffer, Kofoed-Enevoldsen Allan, Cantor Kenneth P., Brinton Louise A. (2002). Hyperparathyroidism and subsequent cancer risk in Denmark. Cancer.

[9] Kissin M, Bakst H (1947). Co-existing myxedema and hyperparathyroidism: case report. J Clin Endocrinol.

[10] Klyachkin M, Sloan D (2001). Secondary hyperparathyroidism: evidence for an association with papillary thyroid cancer. Am Surg.

[11] Tarrass F, Daki S, Benjelloun M, Ramdani B, Garbi Benghanem M, Zaid D (2005). Synchronous papillary thyroid carcinoma and secondary hyperparathyroidism: report of cases and review of the literature. Oral Oncol Extra.

[12] Kaptein EM (1996). Thyroid hormone metabolism and thyroid diseases in chronic renal failure. Endocr Rev.

[13] Miki H, Oshimo K, Inoue H, Kawano M, Morimoto T, Monden Y (1992). Thyroid carcinoma in patients with secondary hyperparathyroidism. J Surg Oncol.

[14] Linos DA, van Heerden JA, Edis AJ (1982). Primary hyperparathyroidism and nonmedullary thyroid cancer. Am J Surg.

[15] Walker MD, Silverberg SJ (2017). Primary hyperparathyroidism. Nat Rev Endocrinol.

[16] Pitt SC, Sippel RS, Chen H (2009). Secondary and tertiary hyperparathyroidism, state of the art surgical management. Surg Clin North Am.

[17] Kutluturk K, Otan E, Yagci MA, Usta S, Aydin C, Unal B (2014). Thyroid pathologies accompanying primary hyperparathyroidism: a high rate of papillary thyroid microcarcinoma thyroid pathologies accompanying primary hyperparathyroidism: a high rate of papillary thyroid microcarcinoma. Turkish J Surg.

[18] Zheng Y, Xu S, Wang P, Chen L (2007). Preoperative localization and minimally invasive management of primary hyperparathyroidism concomitant with thyroid disease. J Zhejiang Univ Sci B.

[19] Masatsugu T, Yamashita H, Noguchi S, Nishii R, Watanabe S, Uchino S (2005). Significant clinical differences in primary hyperparathyroidism between patients with and those without concomitant thyroid disease. Surg Today.

[20] Kösem M, Algün E, Kotan Ç, Harman M, Öztürk M (2004). Coexistent thyroid pathologies and high rate of papillary cancer in patients with primary hyperparathyroidism: controversies about minimal invasive parathyroid surgery. Acta Chir Belg.

[21] Bentrem DJ, Angelos P, Talamonti MS, Nayar R (2002). Is preoperative investigation of the thyroid justified in patients undergoing parathyroidectomy for hyperparathyroidism?. Thyroid..

[22] Sidhu S, Campbell P (2001). Thyroid pathology associated with primary hyperparathyroidism. Aust N Z J Surg.

[23] Burmeister L, Sandberg M, Carty S, Watson C (1996). Thyroid carcinoma found at parathyroidectomy. Cancer..

[24] Attie J, Vardhan R (1993). Association of hyperparathyroidism with nonmedullary thyroid carcinoma: review of 31 cases. Head Neck.

[25] Gates JD, Benavides LC, Shriver CD, Peoples GE, Stojadinovic A (2009). Preoperative thyroid ultrasound in all patients undergoing parathyroidectomy?. J Surg Res.

[26] Trout H, Mulder D (1972). Surgery for parathyroid adenoma. Arch Surg.

[27] Ellenberg AH, Goldman L, Gordon GS, Lindsay S (1962). Thyroid carcinoma in patients with hyperparathyroidism. Surgery..

[28] Monroe DP, Edeiken-Monroe BS, Lee JE, Evans DB, Perrier ND (2008). Impact of preoperative thyroid ultrasonography on the surgical management of primary hyperparathyroidism. Br J Surg.

[29] Krause U, Olbricht T, Metz K, Rudy T, Benker G (1991). Coincidence of non-medullary thyroid cancer and hyperparathyroidism. Chirurg..

[30] Feind CR (1964). Re-exploration for parathyroid adenoma. Am J Surg.

[31] Beus KS, Stack BC (2004). Synchronous thyroid pathology in patients presenting with primary hyperparathyroidism. Am J Otolaryngol.

[32] Miyauchi Akira, Ito Yasuhiro, Oda Hitomi (2018). Insights into the Management of Papillary Microcarcinoma of the Thyroid. Thyroid.

[33] Jovanovic MD, Zivaljevic VR, Diklic AD, Rovcanin BRV, Zoric G, Paunovic IR (2017). Surgical treatment of concomitant thyroid and parathyroid disorders: analysis of 4882 cases. Eur Arch Oto-Rhino-Laryngology.

[34] Burmeister L, Sandberg M, Carty S, Watson C (1996). Thyroid carcinoma found at parathyroidectomy. Association with primary, secondary, and tertiary hyperparathyroidis. Cancer..

[35] Krementz ET, Yeager R, Hawley W, Weichert R (1971). The first 100 cases of parathyroid tumor from Charity Hospital of Louisiana. Ann Surg.

[36] Cuhaci N, Ozdemir D, Polat B, Arpacı D, Yıldırım N, Yazgan AK (2017). Concomitant thyroid lesions in patients with primary hyperparathyroidism. Asian J Surg.

[37] Emirikci S, Ozcinar B, Oner G, Omarov N, Agcaoglu O, Soytas Y (2015). Thyroid cancer incidence in simultaneous thyroidectomy with parathyroid surgery. Ulus Cerrahi Derg.

[38] Coculescu M, Ursu H. Endemic goiter and iodine deficiency disorders. Coll Physicians from Rom Guidel Pract Med Bucharest InfoMedica. 2001;:119–52.

[39] Ruda JM, Hollenbeak CS, Stack BC (2005). A systematic review of the diagnosis and treatment of primary hyperparathyroidism from 1995 to 2003. Otolaryngol Neck Surg.

[40] Lin SD, Te Tu S, Hsu SR, Chang JHM, Yang KT, Yang LH (2005). Synchronous parathyroid and papillary thyroid carcinoma. J Chinese Med Assoc.

[41] Walgenbach S, Hommel G, Junginger T (2000). Outcome after surgery for primary hyperparathyroidism: ten-year prospective follow-up study. World J Surg.

[42] Savli H, Sevinc A, Sari R, Ozen S, Buyukberber S, Ertas E (2001). Occult parathyroid carcinoma in a patient with papillary thyroid carcinoma and Hashimoto’s thyroiditis. J Endocrinol Investig.

[43] Schoretsanitis G, Melissas J, Kafousi M (2002). Synchronous parathyroid and papillary thyroid carcinoma: a case report. Am J Otolaryngol.

[44] Lever E, Refetoff S, Straus F, Nguyen M, Kaplan E (1983). Coexisting thyroid and parathyroid disease - are they related?. Surgery..

[45] Kaplan L, Katz A, Ben-Isaac C, Massry S (1971). Malignant neoplasms and parathyroid adenoma. Cancer..

[46] Boehm BO, Rosinger S, Belyi D, Dietrich JW (2011). The parathyroid as a target for radiation damage. N Engl J Med.

[47] Kaminskyi O, Kopylova O, Afanasyev DY, Mazurenko OV, Berezovskyi SY (2017). Pilot study of parathyroid glands in adult and pediatric subjects exposed to ionizing radiation after the ChNPP accident, methodology of parathyroid diagnostic ultrasound. Probl Radiatsiinoi Medytsyny ta Radiobiolohii.

[48] Fujiwara S, Sposto R, Shiraki M, Yokoyama N, Sasaki H, Kodama K (1994). Levels of parathyroid hormone and calcitonin in serum among atomic bomb survivors. Radiat Res.

[49] Song E, Han M, Oh H-S, Kim WW, Jeon M, Lee Y (2019). Lobectomy is feasible for papillary thyroid carcinomas sized 1–4 cm: a 10-year propensity score matched pair analysis on recurrence. Thyroid..

[50] Calò PG, Conzo G, Raffaelli M, Medas F, Gambardella C, De Crea C (2017). Total thyroidectomy alone versus ipsilateral versus bilateral prophylactic central neck dissection in clinically node-negative differentiated thyroid carcinoma. A retrospective multicenter study. Eur J Surg Oncol.

[51] Conzo G, Polistena A, Calò PG, Bononi P, Gambardella C, Mauriello C (2014). Efficacy of combined treatment for anaplastic thyroid carcinoma: results of a multinstitutional retrospective analysis. Int J Surg.

[52] Morita SY, Somervell H, Umbricht CB, Dackiw APB, Zeiger MA (2008). Evaluation for concomitant thyroid nodules and primary hyperparathyroidism in patients undergoing parathyroidectomy or thyroidectomy. Surgery..

[53] Apetrii M, Goldsmith D, Nistor I, Siriopol D, Voroneanu L, Scripcariu D (2017). Impact of surgical parathyroidectomy on chronic kidney disease-mineral and bone disorder (CKD-MBD) – a systematic review and meta-analysis. PLoS One.

[54] Ito Y, Miyauchi A, Kihara M, Higashiyama T, Kobayashi K, Miya A (2014). Patient age is significantly related to the progression of papillary microcarcinoma of the thyroid under observation. Thyroid..

[55] Ito Y, Kakudo K, Hirokawa M, Fukushima M, Tomoda C, Inoue H (2009). Clinical significance of extrathyroid extension to the parathyroid gland of papillary thyroid carcinoma. Endocr J.

[56] Eigelberger MS, Clark OH (2000). Surgical approaches to primary hyperparathyroidism. Endocrinol Metab Clin N Am.

[57] Prager G, Riss P, Bieglmayer C, Niederle B (2003). The role of intraoperative quick PTH measurements in primary hyperparathyroidism. Ann Ital Chir.

[58] Lee CR, Park S, Kang SW, Lee J, Jeong JJ, Nam KH (2017). Is familial papillary thyroid microcarcinoma more aggressive than sporadic form?. Ann Surg Treat Res.

[59] Diaconescu M, Glod M, Costea I, Grigorovici M, Covic A, Diaconescu S (2011). Surgical management of renal hyperparathyroidism: a preliminary series report. Chir..

[60] Seehofer D, Rayes N, Klupp J, Nüssler NC, Ulrich F, Graef K-J (2005). Prevalence of thyroid nodules and carcinomas in patients operated on for renal hyperparathyroidism: experience with 339 consecutive patients and review of the literature. World J Surg.

[61] Ogburn P, Black B (1956). Primary hyperparathyroidism and papillary adenocarcinoma of the thyroid; report of four cases. Proc Staff Meet Mayo Clin.

[62] Cinamon U, Levy D, Marom T (2015). Is primary hyperparathyroidism a risk factor for papillary thyroid Cancer ? An exemplar study and literature review. Int Arh Otorhinolaryngol.

[63] Wright MC, Jensen K, Mohamed H, Drake C, Mohsin K, Monlezun D (2017). Concomitant thyroid disease and primary hyperparathyroidism in patients undergoing parathyroidectomy or thyroidectomy. Gland Surg.

[64] Ryan S, Courtney D, Timon C (2014). Co-existent thyroid disease in patients treated for primary hyperparathyroidism: implications for clinical management. Eur Arch Otorhinolaryngol.

[65] Thakker R (2016). Genetics of parathyroid tumours. J Intern Med.

[66] Cappola A, Mandel S (2013). Molecular testing in thyroid cancer: BRAF mutation status and mortality. JAMA..

[67] Bounacer A, Wicker R, Caillou B, Cailleux AF, Sarasin A, Schlumberger M (1997). High prevalence of activating ret proto-oncogene rearrangements, in thyroid tumors from patients who had received external radiation. Oncogene..

[68] Livolsi VA, Feind CR (1976). Parathyroid adenoma and nonmedullary thyroid carcinoma. Cancer..

[69] Prinz R, Barbato A, Braithwaite S, Brooks M, Lawrence A, Paloyan E (1982). Prior irradiation and the development of coexistent differentiated thyroid cancer and hyperparathyroidism. Cancer..

[70] Nishiyama R, Farhi D, Thompson N (1979). Radiation exposure and the simultaneous occurrence of primary hyperparathyroidism and thyroid nodules. Surg Clin North Am.

[71] Uhliarova B, Hajtman A (2018). Hashimoto’s thyroiditis – an independent risk factor for papillary carcinoma. Braz J Otorhinolaryngol.

[72] Takano T (2017). Natural history of thyroid cancer. Endocr J.

[73] Kaliszewski K, Zubkiewicz-Kucharska A, Wojtczak B, Strutyńska-Karpińska M (2016). Multi- and unifocal thyroid microcarcinoma: are there any differences?. Adv Clin Exp Med.

[74] Bradley NL, Wiseman SM (2017). Papillary thyroid microcarcinoma: the significance of high risk features. BMC Cancer.

[75] Dideban S, Abdollahi A, Meysamie A, Sedghi S, Shahriari M (2016). Thyroid papillary microcarcinoma: etiology, clinical manifestations, diagnosis, follow-up, histopathology and prognosis. Iran J Pathol.

